# Seafood Processing and Safety

**DOI:** 10.3390/foods5020034

**Published:** 2016-05-13

**Authors:** Michael L. Jahncke

**Affiliations:** Virginia Tech, Virginia Seafood Agricultural Research and Extension Center, 102 S. King St., Hampton, VA 23669, USA; mjahncke@vt.edu; Tel.: +01-757-727-4861

Food microorganisms are found on all surfaces (skin and gills) and in the intestines of fishery products. There are several different types of spoilage and pathogenic bacteria associated with fishery products. Spoilage bacteria are generally harmless, but can cause changes in the color, flavor, odor, texture and reduce the shelf-life of these products. In contrast, pathogenic bacteria are illness-causing organisms that can produce toxins or cause infections. Controlling these microorganisms requires diligence and attention to detail. Reduced shelf-life, quality deterioration and in some instances, the safety of fishery products, is also associated with chemical/enzymatic changes. Quick cooling/quick freezing and stable low storage temperatures will ensure food safety and slow deleterious changes in texture, color and flavor due to bacterial growth and chemical/biochemical activity.

The first step to ensure safety and to produce maximum product quality is to follow Best Management Practices (BMPs) on commercial fishing vessels, at aquaculture farms, during harvest, handling/processing, storage, and shipping. Currently, most BMPs are voluntary programs, although more and more companies and countries are requiring aquaculture farms and harvest vessels to implement BMPs. On the food safety side, the Hazard Analysis Critical Control Point (HACCP) concept is a risk management food safety program for food processors to prevent, eliminate, or reduce to an acceptable level potential food safety hazards that may be present in fishery products [1]. The support programs for HACCP are Good Manufacturing Practices (GMPs) and Sanitation Standard Operating Procedures (SSOPs) [2]. The Food and Agriculture Organization (FAO) and the World Health Organization (WHO) have incorporated the HACCP and GMP programs into a Code of Practice for Fish and Fishery Products. This code provides “guidance and recommendations on the growing, harvesting, handling, storage, transportation and retail sale of fish, shellfish and aquatic invertebrates” [3].

In this Special Issue on Seafood Processing and Safety, we are addressing five different topics from a USA perspective. Brett Koonse gives an overview on US FDA regulatory HACCP requirements for imported seafood products. Koonse points out that exporting countries and foreign companies must understand the food regulatory requirements of the importing country [4]. Chengchu Liu *et al*. studied how refrigerated and frozen storage affects the growth or survival of *Salmonella* Newport 6962 and *Listeria monocytogenes* Scott A, M0507 and SFL0404 on raw (sashimi) Yellow Fin Tuna (*Thunnus albacares*) [5]. Their studies show that both *Salmonella* and *L. monocytogenes* numbers gradually decline during frozen storage at −18 °C. However, during refrigerated storage (5–7 °C) *Salmonella* numbers declined, but *L. monocytogenes* numbers increased. This illustrates the importance of ensuring that GMPs, SSOPs and HACCP Programs are followed throughout the process to ensure the safety of fishery products. Doris Hicks explains the consumer’s role in seafood safety from purchase through preparation and storage. She points out that it is ultimately the consumer’s responsibility to carefully and properly handle and prepare fishery products in order to ensure the safety and quality of these products [6]. Dewitt and Oliveira provide detailed handling and quality guidelines for fresh fish in modified atmosphere (MA) packaging systems. This review provides details on how MA systems can be used to maintain safety and quality, and increase shelf life of fishery products [7]. Finally, George Baker summarizes and discusses current information on post-harvest processing (PHP) methods to help ensure the safety and quality of bivalve molluscan shellfish. [8].

The findings from these papers illustrate that fishery products are generally safe when handled properly from boat or farm to plate, but they are also highly perishable. Many procedures used to ensure food safety can also contribute to higher quality fishery products and longer shelf-life of these products. After harvest, product safety and quality can be ensured by reducing or eliminating microbial activity, and retarding quality-degrading chemical reactions. The three Ps (*i.e.*, Product Characteristics/Processing Methods/Package Types) have a profound effect on the quality and shelf life of chilled and frozen fishery products.
